# 1438. Latent tuberculosis infection (LTBI) in hematopoietic stem cell transplantation (HSCT) recipients: a retrospective Italian cohort study in Tor Vergata University Hospital, Rome.

**DOI:** 10.1093/ofid/ofac492.1267

**Published:** 2022-12-15

**Authors:** Mirko Compagno, Assunta Navarra, Laura Campogiani, Luigi Coppola, Rossi Benedetta, Iannetta Marco, Delia Goletti, Loredana Sarmati

**Affiliations:** Tor Vergata Hospital, Rome, Lazio, Italy; INMI L.Spallanzani, Rome, Lazio, Italy; Tor Vergata University of Rome, Rome, Lazio, Italy; Tor Vergata Hospital, Rome, Lazio, Italy; Tor Vergata Hospital, Rome, Lazio, Italy; University of Rome Tor Vergata, Rome, Lazio, Italy; INMI L.Spallanzani, Rome, Lazio, Italy; Tor Vergata University of Rome, Rome, Lazio, Italy

## Abstract

**Background:**

Patients undergoing haematopoietic stem cell transplantation (HSCT) have severe and prolonged immunodeficiency and the incidence of tuberculosis (TB) disease in those patients is 10 to 40 times higher than the general population

**Methods:**

This retrospective study performed at Tor Vergata University Hospital in Rome (Italy) included all adult patients who underwent a HSCT in the Hematologic Transplant Unit from January 2015 to December 2019. Data on TB screening, performed through interferon-γ release assay (IGRA), were collected; patients were observed for TB reactivation in a follow up period of at least 3 years

**Results:**

323 HSCT recipients were enrolled in this study. Patients’ characteristics are summarized in table 1. 63 patients (19.5%) were not screened for TB, of which 6.3% were born overseas. 260 HSCT recipients (80.5%) were screened: 228 patients (87.7%) had a negative IGRA result, 21 (8.1%) tested positive, and 11 (4.2%) had an indeterminate result. 98.5% were screened before the HSCT. Of the 21 patients with a positive pre-HSCT IGRA test, 14 (66.7%) were males and significantly (p< 0.001) older (median age 60 [IQR] 58-65 years) than those with negative or indeterminate results. Almost all patients (95.2%) were Italian. Patients with a positive IGRA showed a higher monocyte count compared to the other groups (p=0.044), while those with indeterminate IGRA showed a tendence to lower lymphocyte absolute counts, compared to the other groups (p=0.060) (Table 2).

All the 21 patients with a pre-HSCT positive IGRA test were radiologically and clinically screened for active TB and, once it was ruled out, treated for latent TB (LTBI) with isoniazid (INH) for 6 months. No events of toxicity or treatment interruption were reported. None of the patients was treated with rifampin. None of the 21 patients with pre-HSCT positive IGRA test developed active TB during the follow up period.

**Table 1.**
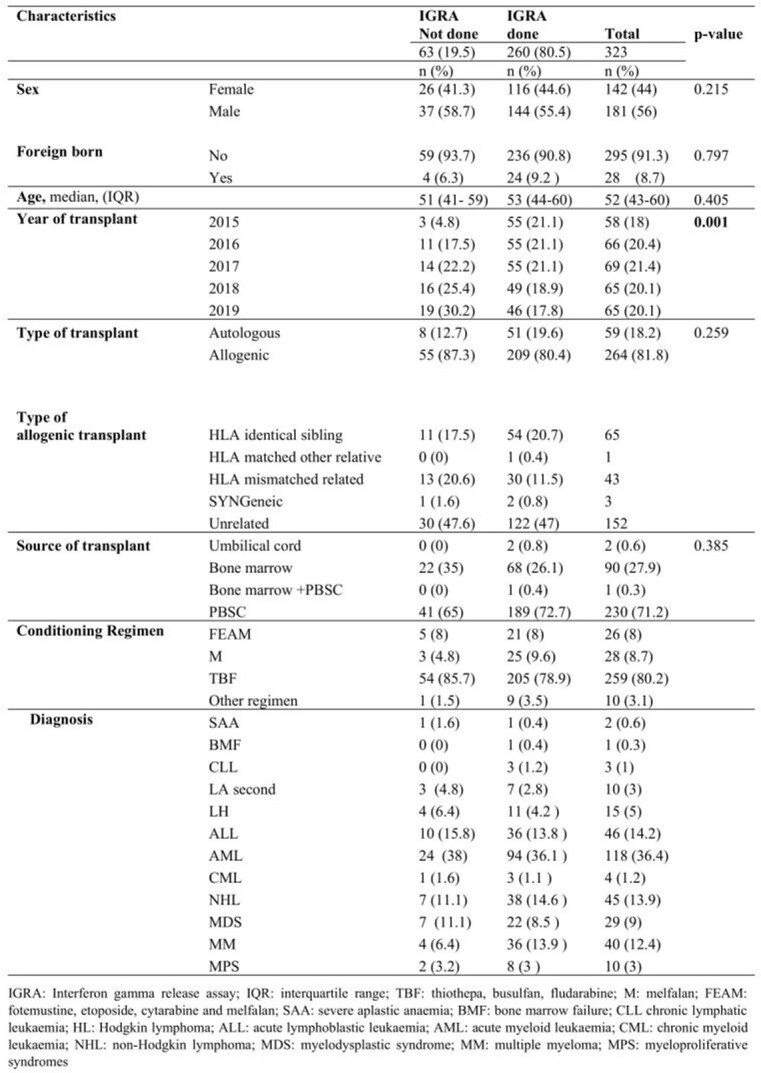
Characteristics of the study population, overall and divided according to IGRA testing

**Table 2.**
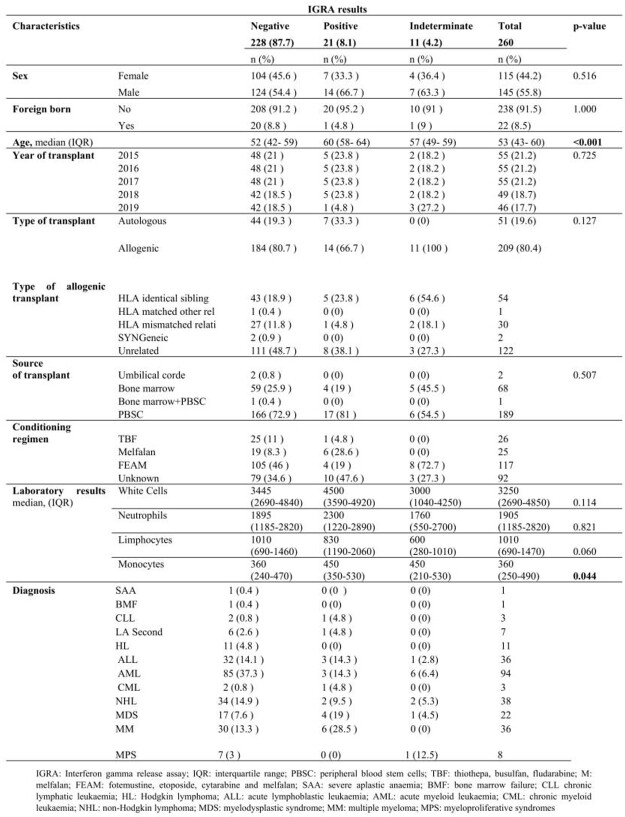
Characteristics of the IGRA tested patients, overall and divided by IGRA test results

**Conclusion:**

To our knowledge, this is the first Italian study on the prevalence of LTBI in patients undergoing HSCT. 8.1% of the 260 patients screened with an IGRA tested positive. The great majority (95.2%) of LTBI patients were Italian and significantly older than IGRA-negative patients. All LTBI HSCT patients received INH treatment and no drug toxicity, nor TB reactivation was observed during the follow-up period

**Disclosures:**

**All Authors**: No reported disclosures.

